# Cofilin 1 promotes bladder cancer and is regulated by TCF7L2

**DOI:** 10.18632/oncotarget.20664

**Published:** 2017-09-06

**Authors:** Fei Wang, Dinglan Wu, Housheng Fu, Fengrong He, Congjie Xu, Jiaquan Zhou, Daoyuan Li, Guoping Li, Jianbing Xu, Qinghui Wu, Jianxiang Chen, Liangju Su, Weifu Wang, Shufang Zhang

**Affiliations:** ^1^ Department of Urology, People's Hospital of Hainan Province, Haikou, China; ^2^ The Clinical Innovation & Research Center (CIRC), Shenzhen Hospital, Southern Medical University, Shenzhen, China; ^3^ Central Laboratory, Haikou People's Hospital, Central South University Xiangya School of Medicine Affiliated Haikou Hospital, Haikou, China

**Keywords:** bladder cancer, Cofilin 1, transcription factor 7-like 2 (TCF7L2), depolymerizing factor (ADF), luciferase assay

## Abstract

Earlier reports demonstrated that Cofilin expression is increased in bladder cancer samples, though its function remains unknown. Here, we found that Cofilin 1 expression was higher in bladder cancer tissues than in paracancerous tissues. Overexpression of Cofilin 1 promoted, while Cofilin 1 knockdown inhibited, proliferation, migration, and invasion in the T24 and RT4 bladder cancer cell lines. In addition, Cofilin 1 overexpression increased, while Cofilin 1 knockdown decreased, bladder tumor volumes in mouse xenograft experiments. Transcription factor 7-like 2 (TCF7L2) targeted the promoter of the Cofilin 1 gene, and TCF7L2 knockdown or mutations in the Cofilin 1 promoter dramatically decreased Cofilin 1 transcription. TCF7L2 promoted cell proliferation and migration and increased Cofilin 1 protein levels in RT4 and T24 cells. Thus, TCF7L2 contributed to Cofilin 1-induced promotion of bladder cancer development by binding to the Cofilin 1 promoter and increasing its expression.

## INTRODUCTION

Human bladder cancer is one of the most common malignancies worldwide and has high recurrence rates [1, 2]. Genetic susceptibility, tobacco smoking, occupational exposure to carcinogens (such as aromatic amines and polycyclic aromatic hydrocarbons), dietary factors, and environmental pollution increase the risk of developing bladder cancer [1, 3]. Diagnosis and treatment of bladder cancer is very expensive [4]. Several markers, including nuclear matrix protein 22, telomerase, and epidermal growth factor receptor, have been used to detect bladder cancer [5–7]. However, the specificities and/or sensitivities of these markers are not high enough to detect bladder cancer early or to track its progression [5, 8]. The discovery of novel markers or targets could greatly improve diagnosis and treatment of bladder cancer.

Cofilin, an actin depolymerizing factor (ADF), is expressed in various cells and regulates the formation of actin filaments by mediating polymerization and depolymerization [9]. High levels of reactive oxygen species (ROS), which contribute to cancer development, alter Cofilin expression in vascular smooth muscle [10]. Cofilin also contributes to cancer development and tumor cell invasion and is a marker for breast cancer [11]. Increased Cofilin levels are associated with increases in tumor cell motility and carcinoma progression [12]. Proteomics analysis revealed that Cofilin expression is also increased in bladder cancer samples [8, 13]. Increased expression and phosphorylation of Cofilin might impact the development and invasiveness of bladder cancer as well [14]. In mammals, there are three different ADFs/Cofilins: Cofilin 1, Cofilin 2, and ADF [9]. Cofilin 1 is expressed in most embryonic and adult mammalian cells, Cofilin 2 is expressed in muscle cells, and ADF is restricted to epithelia and endothelia [9]. Cofilin 1, the non-muscle isoform of the *cofilin 1* gene product, plays a key role in cell migration and cytokinesis and is directly associated with the invasion, metastasis, and chemoresistance of various human malignant solid tumors [11, 15, 16, 17]. Protein kinase D1 (PKD 1) enhances phosphorylation and de-phosphorylates Cofilin 1 to maintain it in its inactive form [18, 19]. Transcription factor 7-like 2 (TCF7L2) and β-catenin levels were lower in cells overexpressing PKD 1 than in control cells [20]. β-catenin/TCF7L2 transcription complex levels were also decreased in PKD 1 overexpression cells compared to control cells [20]. The effects of Cofilin 1 on cancer development and progression therefore depend on its activation status, which is regulated by PKD 1 and TCF7L2. We suspect that Cofilin 1 is regulated by TCF7L2 and affects the development of bladder cancer.

TCF7L2 microsatellite instability and the expression of different splice forms occur in human bladder cancer cells, suggesting that the TCF7L2-mediated signal pathway affects bladder cancer progression [21]. TCF7L2, also known as TCF 4, regulates the transcription of numerous genes targeted by the Wnt signaling pathway [22]. Tiling array analysis and ChIP-PCR identified miR-21 as a candidate target of TCF7L2 [22]. miR-21 was up-regulated in bladder cancer tissues [23]. Together, these results suggest that TCF7L2 mediates bladder cancer progression [24]. In addition, the Wnt signaling pathway regulates bladder cancer metastasis by activating matrix metallopeptidase 9 (MMP 9) [25]. Inhibition of Wnt inhibitory factor-1, an inhibitor of the Wnt signaling pathway, is also ubiquitous in bladder cancer [24].

Here, we studied the effects of Cofilin 1 and TCF7L2 on bladder cancer. First, we measured Cofilin 1 expression in bladder cancer and paracancerous tissues and in the T24 and RT4 bladder cancer cell lines. The effects of TCF7L2 on Cofilin 1 protein expression were also examined. We then investigated the effects of Cofilin 1 on cell proliferation, apoptosis, cell cycle progression, adhesion, migration, and invasion. ChIP and luciferase assays were used to identify factors that regulated Cofilin 1 activity. Finally, the functions of Cofilin 1 in bladder cancer were confirmed in animal experiments. The results revealed that Cofilin 1, which was regulated by TCF7L2, promoted bladder cancer development.

## RESULTS

### Cofilin 1 expression in bladder cancer tissues and cells

**Figure 1:**
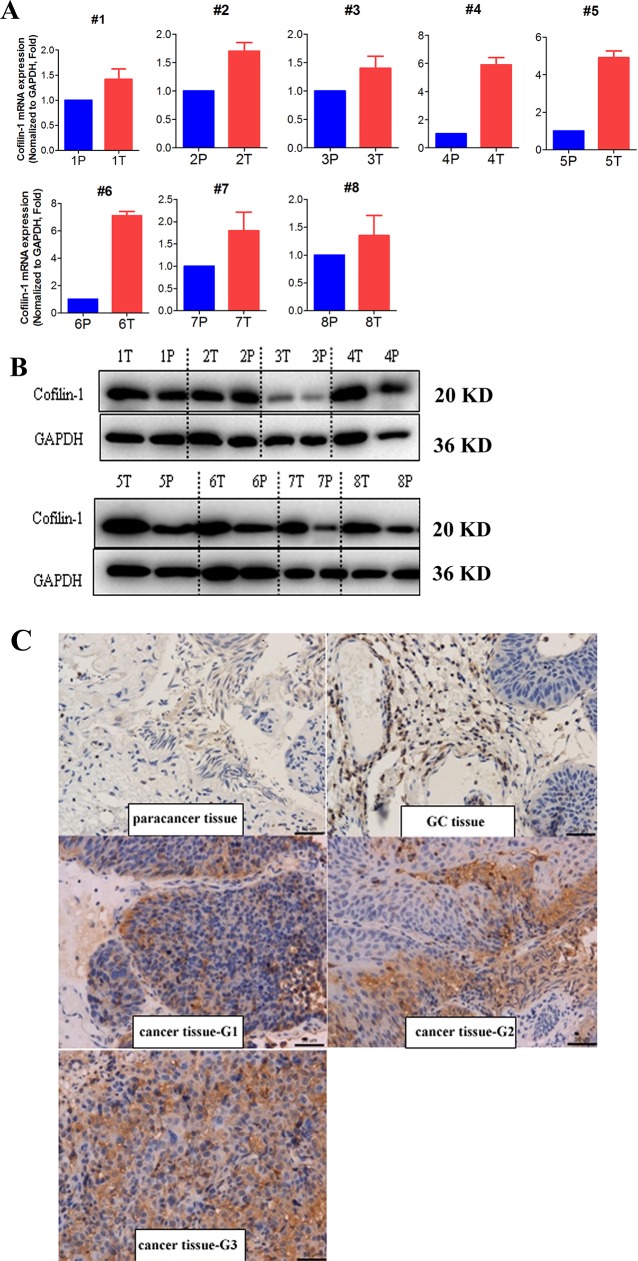
Cofilin 1 expression in bladder cancer and corresponding paracancerous tissues **(A, B)** Cofilin 1 mRNA and protein expression in bladder cancer (T) and paracancerous (P) tissues was detected by RT-PCR and WB. **(C)** Cofilin 1 expression in bladder cancer, glandular cystitis (GC), and paracancerous tissues was detected by WB and IHC.

Cofilin 1 mRNA expression in bladder cancer tissues and corresponding paracancerous tissues as well as T24 and RT4 bladder cancer cells was measured using RT-PCR. Cofilin 1 mRNA expression was higher in bladder cancer tissues than in corresponding paracancerous tissues (Figure 1A). WB and IHC revealed that Cofilin 1 protein levels were higher in bladder cancer tissues than in corresponding paracancerous tissues (Figure 1B and 1C). Cofilin 1 expression in the si-Cofilin 1 group was about 25% of that in control group, indicating that Cofilin 1 knockdown was successful (Supplementary Figure 1). Cofilin 1 expression increased by 100% in cells transfected with Cofilin 1 plasmid compared to the control group (Supplementary Figure 1).

### Cofilin 1 promotes cell growth and proliferation

**Figure 2:**
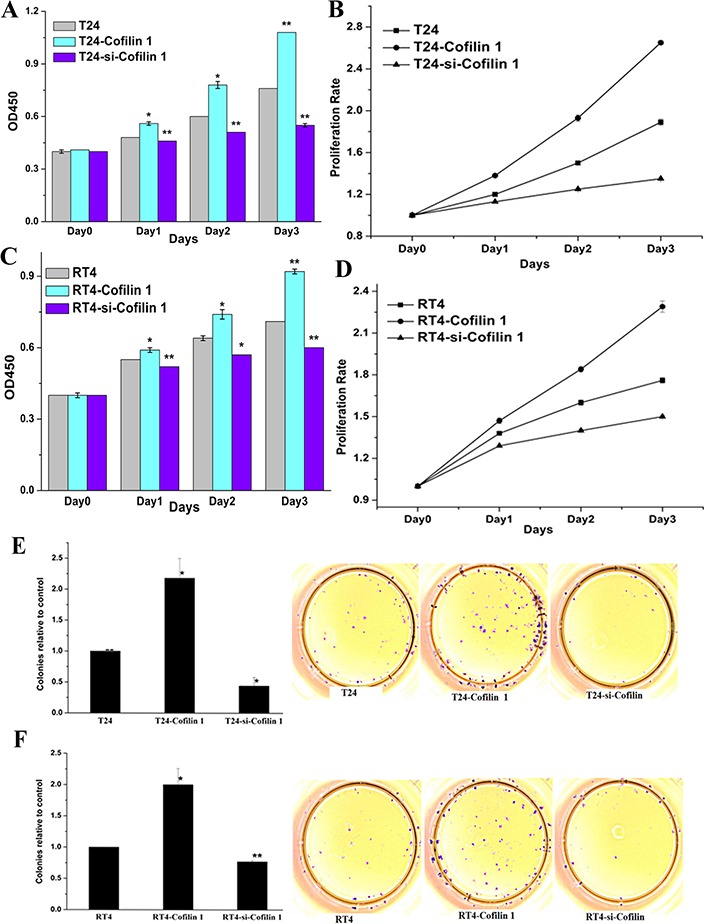
Cofilin 1 promotes cell growth **(A and C)** Effects of Cofilin 1 on cell growth. **(B and D)** Proliferation rates in T24 and RT4 cells transfected with different vectors. The data are presented as means ± SEM. **(E and F)** Cofilin 1 promoted colony formation in T24 and RT4 bladder cancer cell lines. Data shown are representative of three independent experiments, ^*^p < 0.005, ^**^p < 0.001.

To investigate the function of Cofilin in bladder cancer, we transfected cell lines with vector expressing Cofilin 1 or with siRNA to knockdown Cofilin 1. OD450 values were then measured and used to calculate cell proliferation rates (Figure 2A-2D). Cofilin 1 overexpression increased OD450 values and proliferation rates, while Cofilin 1 knockdown decreased OD450 values and inhibited proliferation, in T24 and RT4 cells.

To further investigate the effects of Cofilin 1 on cell growth, the clonogenic capacity of T24 and RT4 cells was evaluated in a colony formation assay (Figure 2E and 2F). Cofilin 1 overexpression increased holoclone establishment in T24 and RT4 cells, while siRNA-induced Cofilin 1 knockdown markedly inhibited clonogenic capacity in both cell lines. These results suggest that Cofilin 1 promotes cell proliferation.

### Cofilin 1 inhibits apoptosis and promotes cell cycle progression

**Figure 3:**
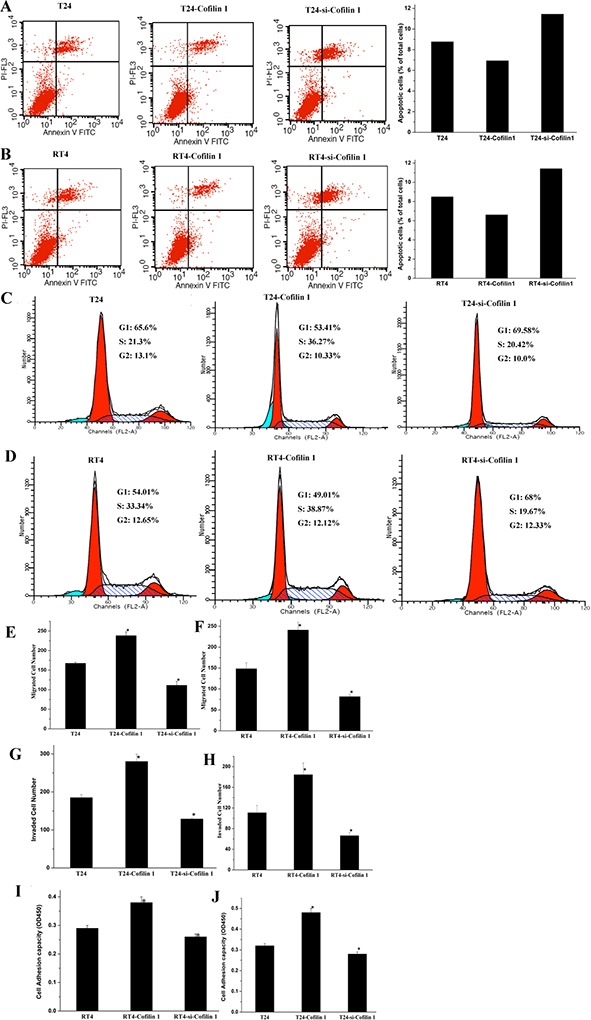
Cofilin 1 inhibits apoptosis and promotes cell cycle progression, migration, invasion, and adhesion **(A and B)** Apoptosis analysis for T24 and RT4 cells transfected with different vectors. Cells were stained with Annexin V-FITC/PI and analyzed by flow cytometry to determine early and late apoptosis cell populations. Percentages of apoptotic cells in each group relative to total cell numbers were used to evaluate apoptosis rates. **(C and D)** Cell cycle population changes in T24 and RT4 cells transfected with different vectors were assessed by flow cytometry using PI staining to measure the DNA content. **(E and F)** Migration and invasion capacity of T24 and RT4 cells transfected with Cofilin 1 and si-Cofilin 1. **(G and H)** Invasion capacity and cell adhesion in T24 and RT4 cells transfected with Cofilin 1 and si-Cofilin 1. **(I and J)** Cell adhesion capacity in T24 and RT4 cells transfected with Cofilin 1 and si-Cofilin 1. The data are presented as the means of three independent experiments, ^*^p < 0.01.

To determine the effects of Cofilin 1 on cell death, apoptosis of T24 and RT4 cells transfected with Cofilin 1 and si-Cofilin 1 vectors was measured using flow cytometry (Figure 3A and 3B). Treatment-dependent shifts in cell populations clearly indicated that apoptosis rates in the si-Cofilin 1-treated group, which increased 1.6-fold compared to the control group, were higher than those of both the Cofilin 1 and control groups. In contrast, Cofilin 1 overexpression decreased the apoptosis rate to 80% of the control group rate. These results suggest that Cofilin 1 knockdown promoted, while Cofilin 1 overexpression inhibited, cell apoptosis.

We then examined the effects of Cofilin 1 on cell cycle progression in T24 and RT4 cells using flow cytometry (Figure 3C and 3D). The G1 phase cell population was lower in Cofilin 1 group than in the control and si-Cofilin 1 groups, suggesting that Cofilin 1 overexpression promoted cell cycle progression. This result was in line with the observation that Cofilin 1 overexpression promoted cell proliferation. In addition, the G1 population was higher in the si-Cofilin 1 group than in the control and Cofilin 1 groups, which indicated that Cofilin 1 knockdown arrested the cell cycle in G1 phase. This result was consistent with the finding that Cofilin 1 knockdown inhibited cell proliferation. Cofilin 1 thus promoted cell proliferation and survival.

### Cofilin 1 promotes cell migration, invasion, and adhesion

Migration and invasion assays were performed to examine the effects of Cofilin 1 on these processes in T24 and RT4 cells. The cell invasion assay was similar to the cell migration assay, but also required cells to migrate through Matrigel to be considered invasive. Numbers of migrated and invaded cells were higher in the Cofilin 1 group than in the control and si-Cofilin 1 groups, which suggests that Cofilin 1 overexpression enhanced cell migration and invasion (Figure 3E-3H and Supplementary Figures 2 and 3). Furthermore, numbers of migrated and invaded cells were lower in the si-Cofilin 1 group than in the control group, which indicated that Cofilin 1 knockdown reduced cellular migration and invasion capacity. Numbers of migrated and invaded cells were higher in all groups for T24 cells than for RT4 cells.

Next, we evaluated the ability of T24 and RT4 cells to adhere to Fibronectin (Fn) (Figure 3I and 3J). Compared to the control group, adhesion capability at 2 hours increased in Cofilin 1 group and decreased in the si-Cofilin 1 group for all cells. These results indicated that Cofilin 1 improved cell adhesion capacities.

### Transcription factor 7-like 2 (TCF7L2) regulates Cofilin 1 expression

**Figure 4:**
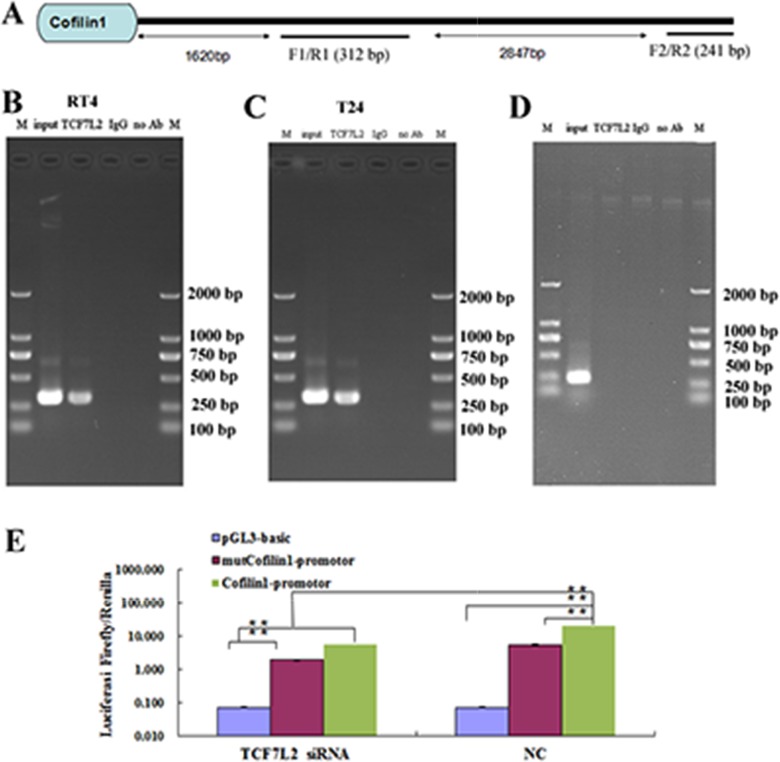
ChIP assay of T24 and RT4 cells **(A)** The sequence of the Cofilin 1 promotor region. **(B and C)** ChIP results detected by PCR with the F1/R1 primer. **(D)** ChIP results detected by PCR with the F2/R2 primer. **(E)** Luciferase assay. Relative luciferase activity was calculated as follows: (Rluc miRNA/Luc miRNA)/ (Rluc no-target/Luc nontarget). TCF7L2 knockdown (si-TCF7L2) or mutCofilin 1-promotor decreased luciferase activity. The data are presented as the means of three independent experiments, ^**^p < 0.01.

We predicted that TCF7L2 bound to the Cofilin 1 promoter region; ChIP and luciferase reporter assays were used to confirm this prediction (Figure 4). The Cofilin 1 promoter sequence is shown in Figure 4A. Two primer pairs were designed targeting different sites (Figure 4A); the results of PCR amplification with the F1/R1 and F2/R2 primers are shown in Figure 4B-4D, respectively. Treatment with the TCF7L2 antibody and the F1/R1 primers resulted in a band of the same size as the input, while no band was observed for samples treated with IgG instead of Ab and the F2/R2 primers. These results indicated that TCF7L2 bound to the promoter region of Cofilin 1. The luciferase reporter assay further confirmed that TCF7L2 regulated Cofilin 1 expression (Figure 4E). Luciferase signal strength did not differ between the pGL3-basis-TCF7L2-siRNA and pGL3-basis-NC groups. However, luciferase signal strength was 3.6 times higher in the Cofilin 1-NC group than in the mutCofilin 1-promoter-NC group, which suggests that the Cofilin 1 promoter indeed induced fluorescein expression. In addition, the luciferase signal strength was 3.6 times higher in the Cofilin 1-NC group than in the Cofilin 1-si-TCF7L2 group, which suggests that siRNA-induced downregulation of TCF7L2 decreased luciferase signal strength. The luciferase signal strength for the mutCofilin 1-promoter-NC group was similar to that of the Cofilin 1-si-TCF7L2 group. Finally, luciferase signal strength in the Cofilin 1-NC and Cofilin 1-si-TCF7L2 groups was 2.8 times that of the mutCofilin 1-promoter-si-TCF7L2 group. Together, these results suggest that TCF7L2 bound to the promoter region of Cofilin 1 and further reduced fluorescein expression.

### TCF7L2 promoted cofilin 1 protein expression

**Figure 5:**
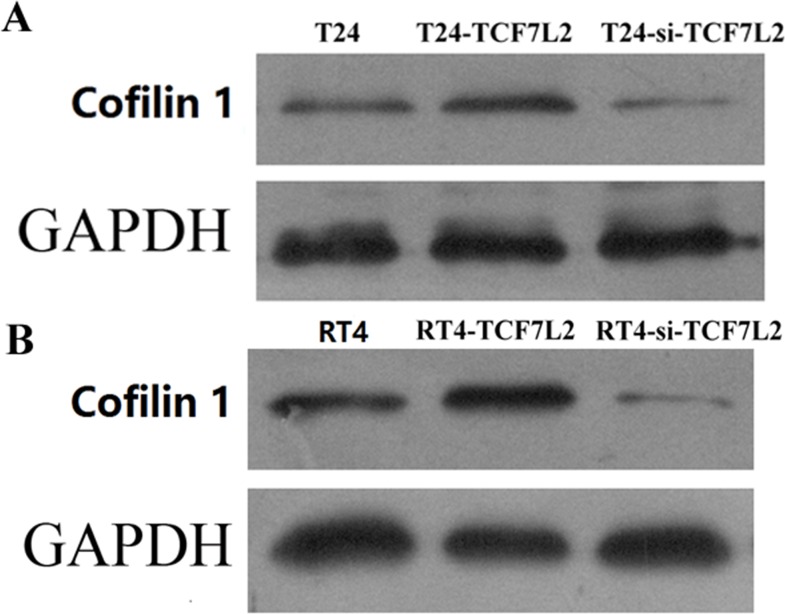
Cofilin 1 protein expression in control, TCF7L2, and si-TCF7L2 group T24 and RT4 cells were detected by WB

As shown in Figure 5, Cofilin 1 protein expression was regulated by TCF7L2 in T24 and RT4 cells. Compared to the control group, Cofilin 1 protein expression was increased in the TCF7L2 group and decreased in the si-TCF7L2 group in both T24 and RT4 cells. These results confirmed that upregulation or downregulation of TCF7L2 resulted in upregulation or downregulation of Cofilin 1, respectively.

### Cofilin 1 promoted bladder tumor growth *in vivo*

**Figure 6:**
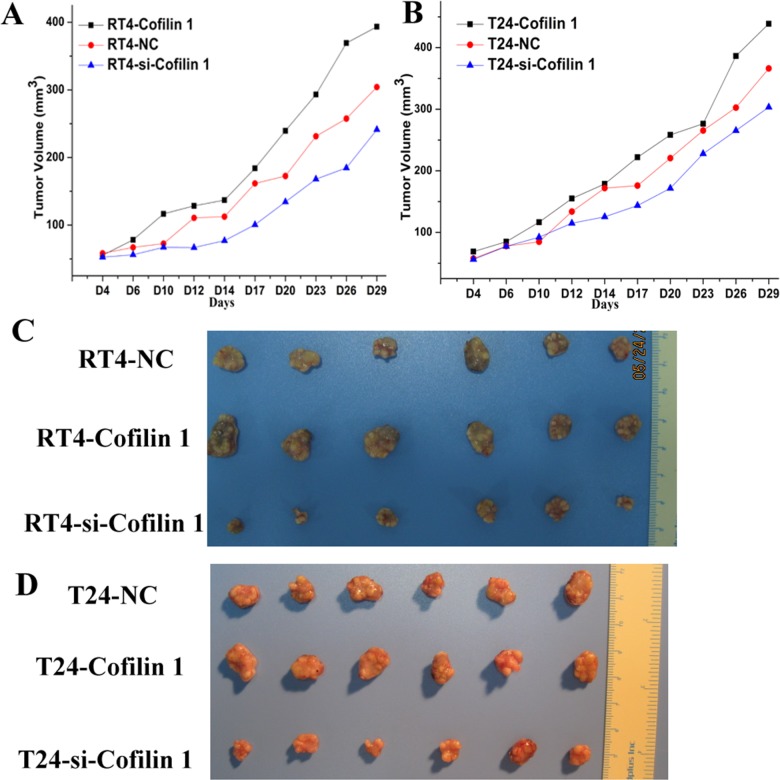
Cofilin 1 promotes bladder tumor growth *in vivo* **(A and B)** Tumor volumes in nude mice (n=6 per group); the data are presented as the means compared to the control group. **(C and D)** Representative photographs of bladder cancer tissues formed after injections of T24 and RT4 transfected with Cofilin 1 or si-Cofilin 1 into nude mice.

To investigate the effect of Cofilin 1 on tumorigenesis, we established a BALB/c nude mouse xenograft model using T24 and RT4 cells. The T24 and RT4 cells were pre-transfected with Cofilin 1, si-Cofilin 1, or NC vectors. These cells were then injected into female BALB/c nude mice to form tumors. Tumor volumes were measured every 2 or 3 days for 29 days (Figure 6A and 6B). Tumor volumes were higher in mice injected with T24 or RT4 cells overexpressing Cofilin 1 than in the control group (NC group). Tumor volumes were lower in mice injected with Cofilin 1 knockdown T24 and RT4 cells (si-Cofilin 1) than that in the control group (NC group). These results indicate that Cofilin 1 increased the tumorigenicity of T24 and RT4 cells in the nude mouse xenograft model. Tumor volumes were larger in mice injected with T24 cells than in the corresponding groups injected with RT4 cells, which further confirmed that T24 cells had stronger migration and invasion capabilities than RT4 cells. Representative photographs of bladder cancer tissues in the nude mice are shown in Figure 6C and 6D. Together, these results suggest that Cofilin 1 indeed promoted bladder cancer formation *in vivo*.

## DISCUSSION

In this study, we found that Cofilin 1 expression was higher in bladder cancer tissues than in paracancerous tissues, which is consistent with previous results [32]. We also examined the effects of Cofilin 1 in the T24 and RT4 bladder cancer cell lines. Cofilin 1 promoted proliferation and decreased apoptosis in T24 and RT4 cells. Furthermore, Cofilin 1 overexpression promoted cell cycle progression, while Cofilin 1 knockdown resulted in G1 phase arrest, which confirmed that Cofilin 1 promoted cell proliferation; however, this result contradicts previous results regarding the effects of Cofilin 1 on the cell cycle [33, 34]. Cofilin 1 enhanced the migration and invasion capacities of T24 and RT4 cells, indicating that Cofilin 1 contributes to invasion and metastasis in bladder tumors as well as in mammary tumors [13, 17, 35]. Cofilin 1 also promoted bladder cancer growth in mouse xenograft experiments. Together, these results and previous studies suggest that Cofilin 1 may be a therapeutic target for bladder cancer [8, 32]. ChIP and luciferase assays demonstrated that the transcription factor TCF7L2 bound to the promoter region of Cofilin 1 to regulate its expression. TCF7L2 knockdown decreased Cofilin 1 protein expression, while upregulation of TCF7L2 increased Cofilin 1 protein expression. In addition, TCF7L2 promoted proliferation and migration in RT4 and T24 cells. Together, these results indicate that Cofilin 1, which is regulated by TCF7L2, promotes bladder cancer development and progression [22, 25, 24]. Cofilin 1 is located at 11q13, a chromosome region that is associated with invasion, metastasis, and decreased survival in cancer patients [36]. Cofilin 1 is phosphorylated by LIM kinase or testicular protein kinase, which results in inactivation, and is dephosphorylated by Cofilin phosphatase Slingshot, which results in reactivation [18, 19, 20, 37]. A previous study demonstrated that Cofilin 1 can transport G-actin and other proteins to the nucleus, which results in changes in gene expression that increase tumor cell proliferation and viability [38]. In addition, Cofilin 1 binds to F-actin and induces remodeling of the actin cytoskeleton, resulting in increased tumor cell motility, invasion, and metastasis [38]. PKD 1 inhibits depolymerization by promoting the phosphorylation or inhibiting the de-phosphorylation Cofilin 1, which maintains it in its inactive form [18, 19]. TCF7L2 and β-catenin levels, as well as β-catenin/TCF7L2 transcription complex levels, are decreased in PKD 1 overexpression cells compared to control cells [20]. Both TCF7L2, which positively regulates Cofilin 1 protein expression and also binds to its own promoter, and Cofilin 1 are regulated by PKD1.

In this study, we confirmed that Cofilin 1 promoted the proliferation, migration, invasion, and adhesion capacities of T24 and RT4 bladder cancer cells while also inhibiting cell apoptosis. In addition, Cofilin 1 increased bladder tumor volumes. Furthermore, we discovered that transcription factor TCF7L2 bound to the Cofilin 1 promoter to increase its expression and promote the development of bladder cancer.

## MATERIALS AND METHODS

### Bladder cancer patient characteristics

We obtained 8 pairs of bladder cancer tissues and paracancerous tissues, which were collected 2 cm away from the bladder cancer tissues, from the hospital. All procedures involving human participants were in performed accordance with the ethical standards of the institutional and/or national research committees and the 1964 Helsinki declaration and its later amendments, or comparable ethical standards.

We retrospectively reviewed data from patients admitted to the 3 institutions included in the study between January and December 2016. Patients more than 18 years old with histologically-proven bladder cancer who had not received any chemotherapy or radiotherapy treatment were included. Patients underwent cystectomy, which included *en bloc* excision of the bladder as well as the prostate and seminal vesicles in men and the uterus, ovaries, and anterior vagina in women. None of the patients had distant metastases in the chest, abdomen, pelvis, or bones at the time of preoperative imaging. Patients who underwent conservative surgery or with different histological subtypes (small cell variants, pure adenocarcinoma, or pure epidermoid carcinoma) were excluded, while patients who received peri-operative chemotherapy were included. Characteristics of the bladder cancer patients are shown in Table 1.

**Table 1:** The clinical information including stages and grades of the bladder cancer patients recruited in this study

	Gender	Bladder cancer (BC) type	WHO phase	TNM staging	Grade	Superfical or invasive
**1**	Female	Papillary urothelial BC	Low	T1N0M0	1	Superfical
**2**	Male	Invasive BC	Medium	T1N0M0	2	invasive
**3**	Male	Non-invasive BC	Low	T1N0M0	1	Superfical
**4**	Male	Invasive BC	High	T2N0M0	3	invasive
**5**	Male	Invasive BC	High	T3N0M0	3	invasive
**6**	Female	Invasive BC	High	T3N0M0	3	invasive
**7**	Male	Invasive BC	Medium	T2N0M0	2	invasive
**8**	Male	Invasive BC	High	T3N0M0	3	invasive

### Reverse transcription polymerase chain reaction (RT-PCR)

Total RNA and protein were extracted from the bladder cancer and paracancerous tissues using an extraction kit (Invitrogen, Carlsbad, CA; Keygen Biotech). For cell experiments, total RNA was extracted using the Trizol method. Cofilin 1 mRNA expression was detected by reverse transcription polymerase chain reaction (RT-PCR). cDNA was synthesized using the PrimeScript II 1st Strand cDNA Synthesis Kit (Takara, Japan). The primer sequences used in the experiment were as follows: Cofilin 1 forward primer: 5′ TTGTGCGGCTCCTACTAA 3′, Cofilin 1 reverse primer: 5′ TTGCATCATAGAGGGCATAG 3′; 18s rRNA forward primer: 5′ CCTGGATACCGCAGCTAGGA 3′, 18s rRNA reverse prime: 5′ GCGGCGCAATACGAATGCCCC. Cofilin 1 mRNA expression was measured via RT-PCR using SYBR Green PC Master Mix (Toyobo, Japan). Thermocycler conditions consisted of initial holds at 50°C for 2 min and 95°C for 5 min followed by a PCR program of 95°C for 15 sec, 60°C for 15 sec, and 72°C for 30 sec for 40 cycles and a final hold at 72°C for 5 sec. Reactions were performed on an ABI PRISM^®^ 7500 Sequence Detection System. Data for all samples was normalized to the control. Cofilin 1 mRNA expression was calculated using the relative quantification equation (RQ=2^−ΔΔCt^) [26].

### Western blotting (WB)

Cofilin 1 protein levels in cancer tissues, paracancerous tissues, and bladder cancer cells were evaluated by Western blotting (WB). Protein concentrations were determined using the BCA Protein Assay Kit (Keygen Biotech). Proteins were detected using a Cofilin 1 monoclonal antibody (ab42824, 1:1000, Abcam, USA) and a GAPDH polyclonal antibody (AP0063, 1: 10,00, Bioworld) and then visualized using a commercial Immobilon Western HRP Substrate (WBKLS0500, Millipore, USA) under dark conditions.

### Immunohistochemistry (IHC)

Cofilin 1 protein expression in bladder cancer tissues and corresponding paracancerous tissues was evaluated by IHC with the 3′, 3′-diaminobenzidine (DAB) kit (ZLI-9017, ZSGB-Bio, Beijing, China), which was performed according to the manufacturer's protocol. Cofilin 1 monoclonal antibody (ab42824, 1:200, Abcam, USA) and goat anti-rabbit HRP antibody (ab136817, 1:400, Abcam, USA) were used.

### Cell culture

The T24 and RT4 human bladder cancer cell lines were obtained from LAND biology company (Guangzhou, China). The cells were cultured using standard procedures and incubated at 37°C with 5% CO_2_. Knockdown of Cofilin 1 and TCF7L2 was performed using small interfering RNA (siRNA). T24 and RT4 cells were transfected with siRNA or Cofilin 1 or TCF7L2 overexpression plasmid vectors using Lipofectamine 2000 (Invitrogen, USA) according to the manufacturer's protocol. Opti-MEM medium (Invitrogen) was used for transfection and was replaced with DEME medium after 4-6 h of incubation.

### Cell proliferation assay

Cell proliferation was measured using the Cell Counting Kit-8 (CCK-8) assay kit (Dojindo, Kumamato, Japan). Absorbance was measured for all samples at 450 nm (OD450). Cell proliferation and inhibition rates were measured from day 0 through day 3 and were calculated based on OD450 values.

### Colony formation assays

The clonogenic assay was performed as previously reported [27]. T24 and RT4 cells were plated at 100 cells/well in a 96-well dishes and incubated for 7 days, after which medium was discarded, cells were washed with PBS 2 times, 200 μL of crystal violet staining solution were added, and cells were then gently washed with water and dried at room temperature. Samples were counted, photographed, and analyzed using the AID EliSpot iSpot Reader System (AID, Germany).

### Cell cycle assay

Cells were cultured for 48 h, then collected, fixed, and stained using standard procedures. The cells were stained with a propidium iodide (PI) solution containing 100 μg/mL PI and 50 μg/mL RNase (Sigma, USA) in PBS at 37°C for 30 min in the dark. After cell clumps were removed by passing stained cells through a nylon mesh sieve, the samples were analyzed by flow cytometry (BD, USA). Data were collected and analyzed using CELL Quest and ModFit LT software.

### Cell apoptosis analysis

Cells were harvested and washed after 48 h of incubation. Cell apoptosis was analyzed using an Annexin V-FITC/PI Apoptosis Detection Kit (Catalog no. KGA106; Nanjing Keygen Biotech Co., Ltd) according to manufacturer's instructions. After staining, all samples were immediately measured on a FACSort flow cytometer (BD, USA). The data were analyzed using Cell Quest 3.0 software (BD, USA).

### Cell adhesion assay

To analyze the adhesion of T24 and RT4 cells to an endothelial monolayer, Fibronectin (Fn) was seeded into a 96-well plate. Cells formed a confluent monolayer within 2 days. Non-adherent cells were removed by washing with PBS. The adherent cells were quantified using the CCK-8 kit as described for the cell proliferation assay.

### Migration and invasion assays

The migration ability of T24 and RT4 cells was assessed using Transwell chambers (BD, REF353097) according to the manufacturer's protocol. Briefly, 1×10^5^ cells were seeded in the upper chamber with serum-free medium. Cells were then allowed to migrate to the lower chamber containing 10% fetal calf serum medium. After non-migratory cells remaining on the upper side of the filter were removed with a cotton swab, migratory cells attached to the underside of the filter were stained with 0.1% crystal violet for 20 min at room temperature. The stain was eluted with 33% acetic acid and cells were photographed after 48 h incubation. The absorbance at 570 nm (OD570) was measured.

Cells incubated in serum-free serum were added to Transwell chambers with a thin layer of Matrigel basement membrane matrix (8 mm pores); 10% fetal calf serum was added to the lower chamber. After noninvasive cells remaining on the upper side of the filter were removed with a cotton swab, invasive cells attached to the underside of the filter were stained with 0.1% crystal violet for 20 min at room temperature. The stain was eluted with 33% acetic acid and cells were photographed after 48 h incubation. The absorbance at 570 nm (OD570) was measured.

### Chromatin immunoprecipitation (ChIP) analysis

TCF7L2 binding sites within the Cofilin 1 promoter were predicted using the Jaspar database (http://jaspar.binf.ku.dk) and then verified using the chromatin immunoprecipitation (ChIP) method [28, 29]. RT4 and T24 cells lysates were subjected to ChIP analysis conducted as previously described [30]. Briefly, 10% of each sample was reserved as input, and the rest was immunoprecipitated with TCF7L2 antibody; isotypic normal IgG was used as a negative control. ChIP was conducted with a ChIP kit (Thermo, 26156). DNA was extracted from the immunoprecipitated samples and subjected to analysis for Cofilin 1 promoter regions by PCR and 2% w/v agarose gel electrophoresis. The reserved input for each sample was used as an internal control. DNA from each sample was used as a template for amplification of the last exon of Cofilin 1 to serve as a PCR negative control; 32 cycles were used, as was the case for the promoter region PCR. The following Cofilin 1 primer pairs were used: forward primer 1: 5′ GCGGGAGAGAAAAGAGTTAACTCC 3′, reverse primer 1: 5′ CAGCACTTCGGAGACGGGCTC 3′; forward primer 2: 5′ ACCCTGTTCCCACCAGTTAGCC 3′, reverse primer 2: 5′ CAAGGCATAAACCTCCAGGAG 3′ as a negative control.

### Luciferase reporter assay

The Cofilin 1 promoter fragment containing putative binding sites for TCF7L2 was obtained and ligated (Takara, Japan) into the digested pGL3 Luciferase reporter vector (Promega, USA) between the XhoI and NotI sites. Cofilin 1 promoter fragment site mutations were generated using the KOD-plus mutagenesis kit (Toyobo, Japan) according to the manufacturer's protocol. RT4 cells were plated in a 96-well plate and then co-transfected with pGL3 basic vector, pGL3-Cofilin 1 promoter, pGL3-mutCofilin 1 vector and siTCF7L2, or NC vector. After 48 h of co-transfection, luciferase activity was detected using the Dual-Glo luciferase assay kit (Promega, USA).

### Xenograft assay

All experimental procedures involving animals were performed according to the Guide for the Care and Use of Laboratory Animals (8th edition) [31]. T24 and RT4 cells were pre-treated with Cofilin 1, si-Cofilin 1, and NC, respectively, for 24 h. The cells were then suspended in 100 μL PBS at 4×10^6^ cells/mL and injected into right flanks of 5-6 week-old BALB/C female athymic nude mice (n = 6 per group). Tumor sizes were monitored by measuring the length (L) and width (W) with calipers, and volumes were calculated using the formula (L×W^2^)/2.

### Statistical analysis

All statistical analyses were performed using Statistical Package for the Social Sciences (SPSS) 19.0 software. The data are presented as the means ± SD of three separate experiments. Statistical significance was determined using paired or unpaired Student's *t*-tests for standardized expression data.

## SUPPLEMENTARY MATERIALS FIGURES



## Abbreviations

ADF: actin depolymerizing factor
TCF7L2: transcription factor 7-like 2
SPSS: Statistical Package for the Social Sciences
ChIP: chromatin immunoprecipitation
Fn: Fibronectin
PI: propidium iodide
CCK-8: Counting Kit-8
DAB: 3′, 3′-diaminobenzidine
WB: Western blotting
MMP-9: metallopeptidase 9
PDK1: Protein kinase D1
