# Supplementary material for: Cofilin 1 promotes bladder cancer and is regulated by TCF7L2
Source: Oncotarget. 2017 Sep 6;8(54):92043–54. doi: 10.18632/oncotarget.20664 (PMC5696162; doi:10.18632/oncotarget.20664)
Supplement: Supplementary file 1 [file oncotarget-08-92043-s001.pdf]

Cofilin 1 promotes bladder cancer and is regulated by TCF7L2

SUPPLEMENTARY MATERIALS

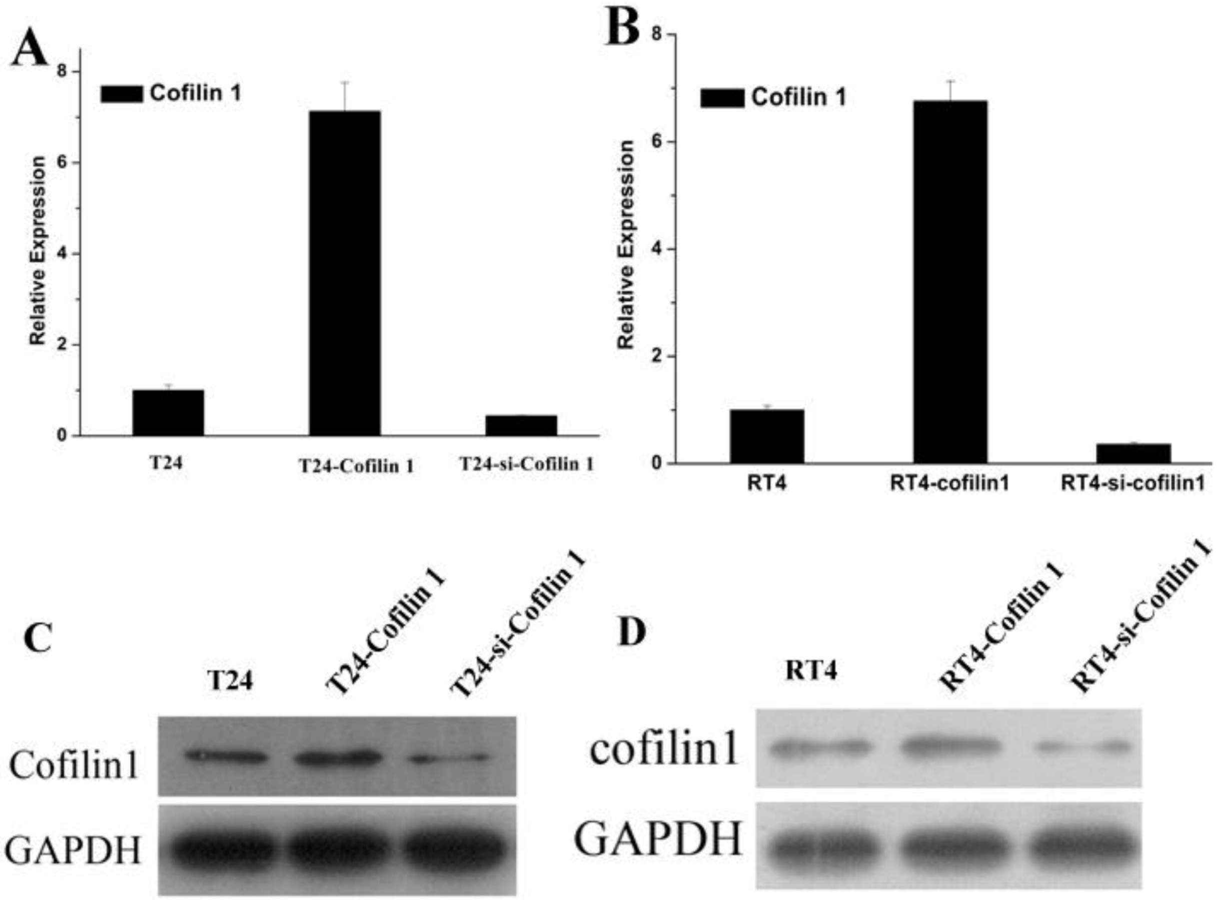

Supplementary Figure 1: Cofilin 1 expression in control, Cofilin 1, and si-Cofilin 1 group T24 **(A, C)** and RT4 **(B, D)** cell lines detected by RT-PCR (A, B) and WB (C, D).

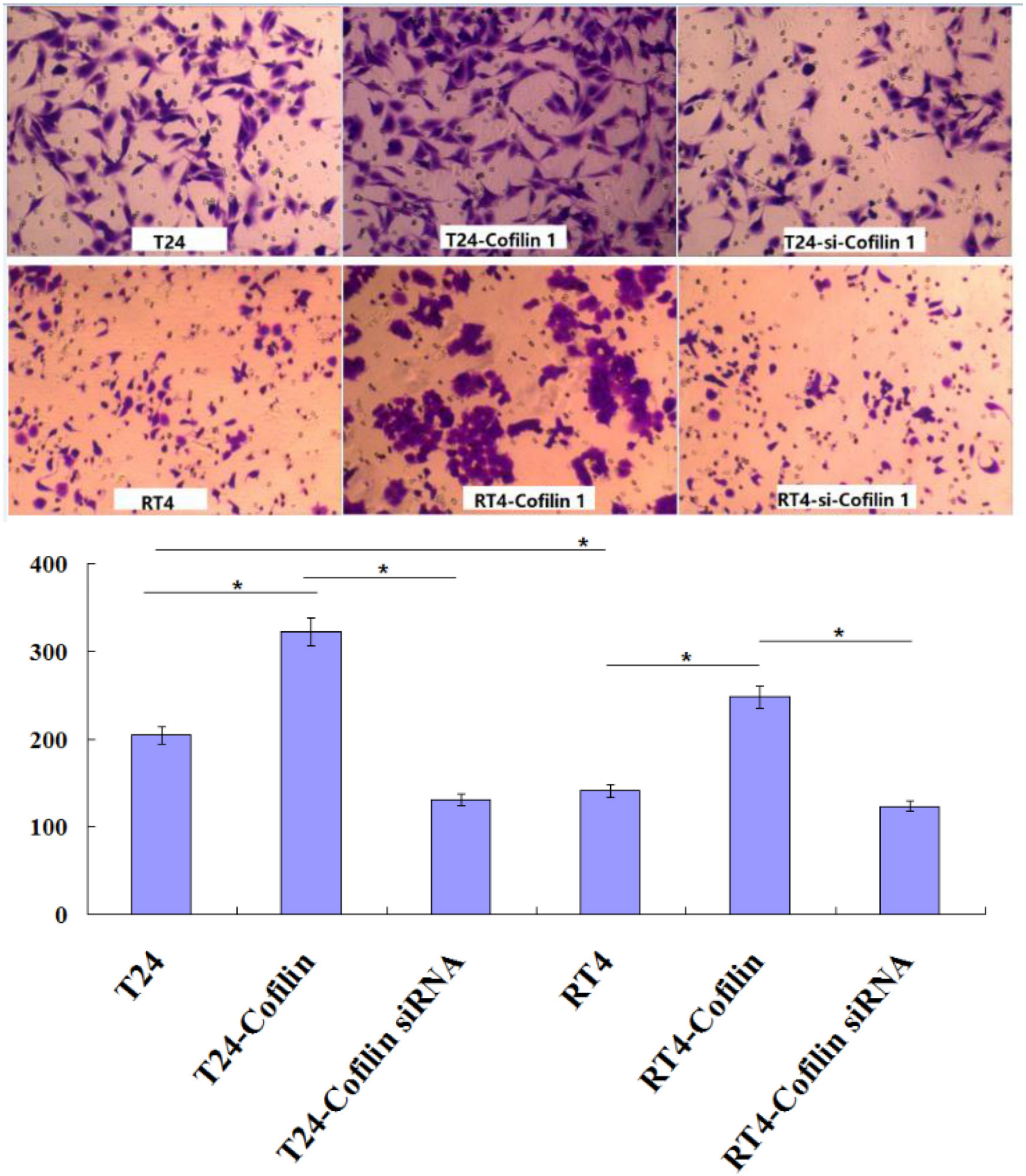

Supplementary Figure 2: Cofilin 1 increased migration capacity in T24 and RT4 cells. Migrated cells were stained with 0.1% crystal violet for 20 min at room temperature. The data are presented as the means of three independent experiments, $*p < 0.05$.

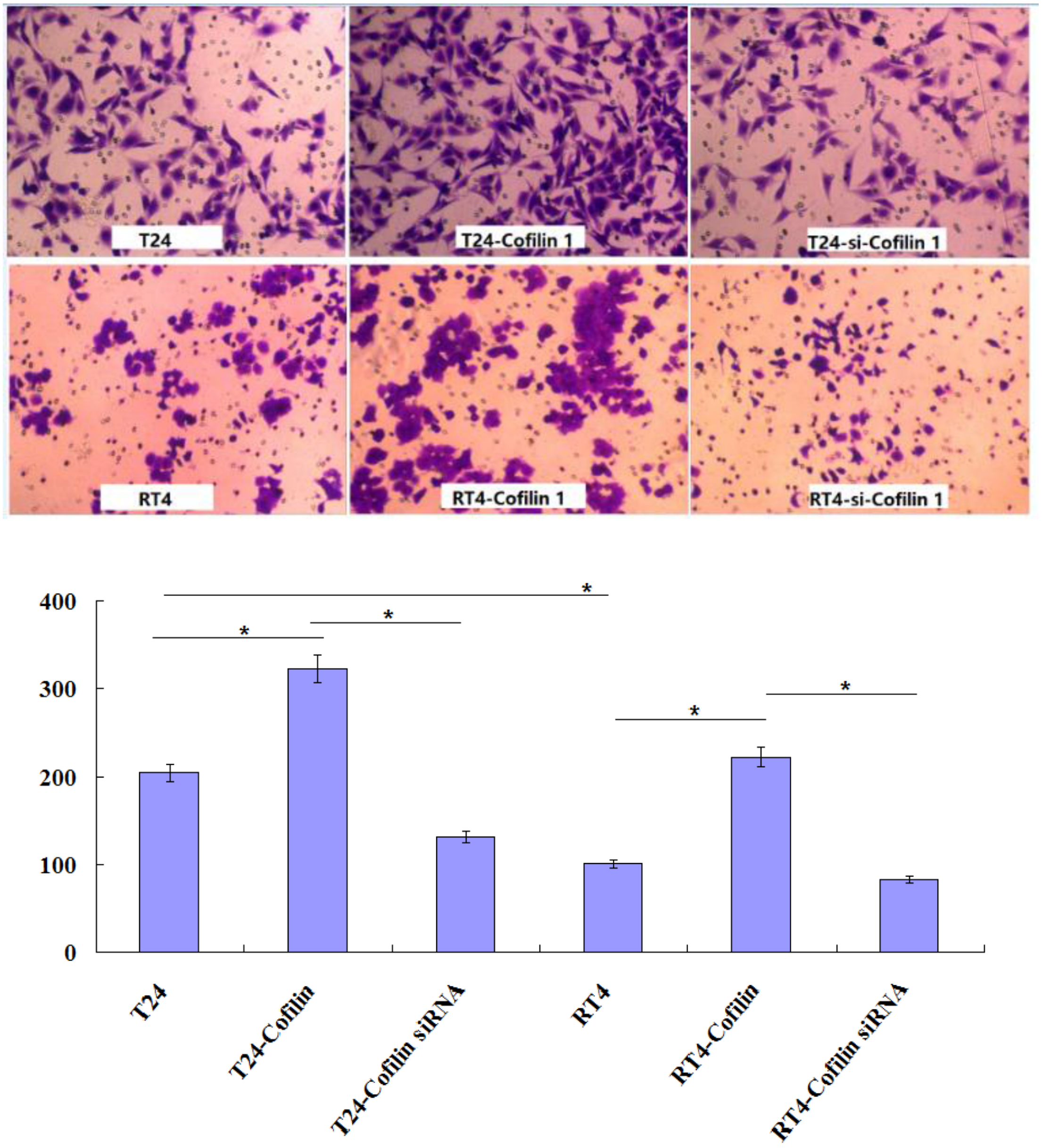

Supplementary Figure 3: Cofilin 1 increased invasion capacity in T24 and RT4 cells. Invaded cells were stained with 0.1% crystal violet for 20 min at room temperature. The data are presented as the means of three independent experiments, $*p < 0.05$.

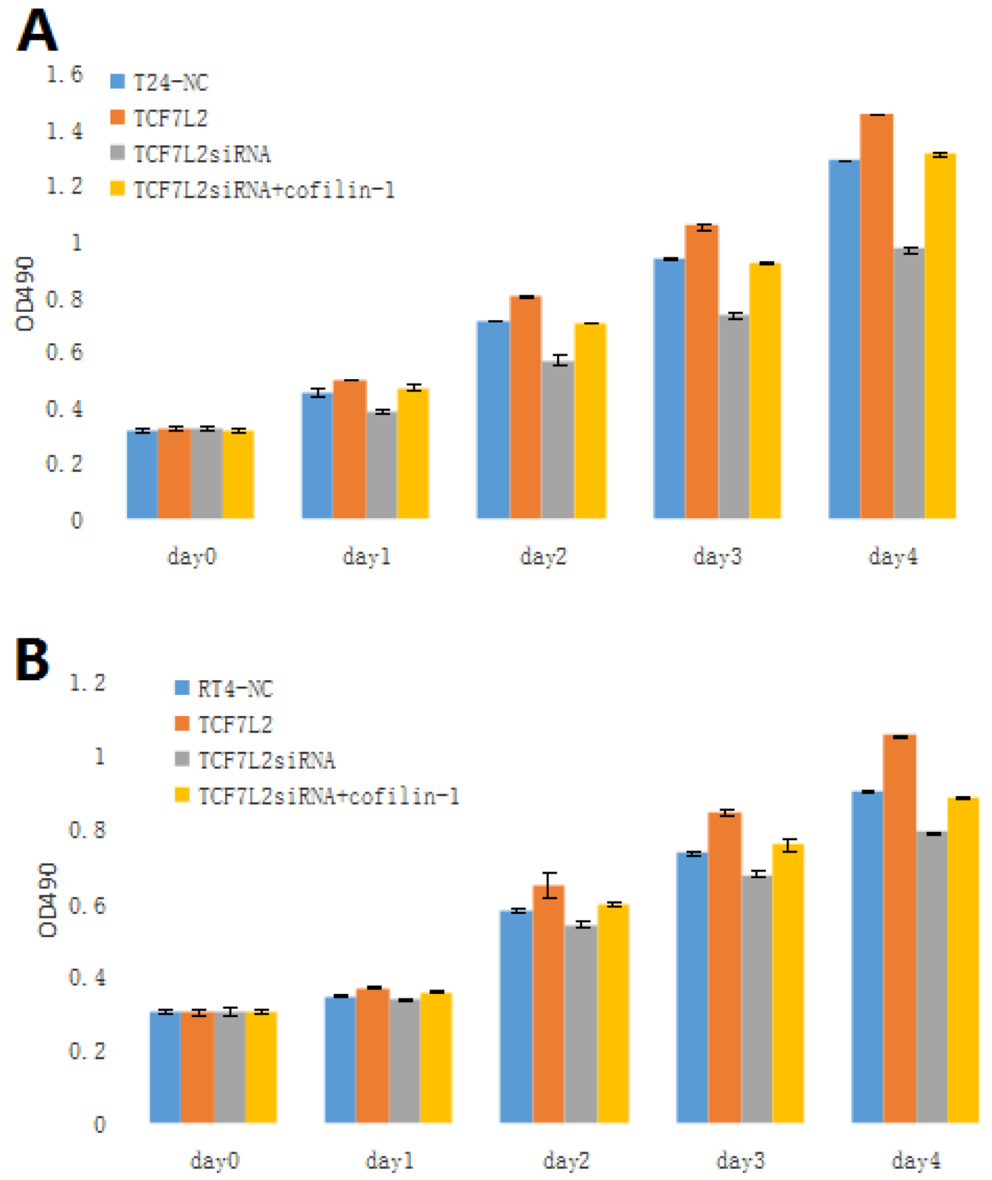

Supplementary Figure 4: Cell proliferation by MTT assay in control, TCF7L2, TCF7L2siRNA, and TCF7L2siRNA+Cofilin 1 group T24 **(A)** and RT4 **(B)** cells. The data are presented as the means of three independent experiments.
